# Initial PCR Testing Negative, but Chest CT Suggesting for Viral Pneumonia Urges for Repeated Testing for COVID-19 Diagnosis

**DOI:** 10.3389/fmolb.2021.640788

**Published:** 2021-05-26

**Authors:** Lingwei Wang, Danting Zhan, Xiaodi Liu, Kai Yang, Shipin Wu, Heng Zhang, Min Yu, Yimin Zha, Weibin Huang, Lei Li, Rongchang Chen, Chen Qiu

**Affiliations:** ^1^ The Department of Respiratory Diseases and Critic Care Unit, Shenzhen Institute of Respiratory Diseases, Shenzhen People’s Hospital (The Second Clinical Medical College, Jinan University; The First Affiliated Hospital, Southern University of Science and Technology), Shenzhen, China; ^2^ Shenzhen Key Laboratory of Respiratory Diseases, Shenzhen Major Respiratory Diseases’s Prevention and Treatment Center, Shenzhen, China; ^3^ Department of Infectious Diseases, Shenzhen People’s Hospital (The Second Clinical Medical College, Jinan University; The First Affiliated Hospital, Southern University of Science and Technology), Shenzhen, China; ^4^ Guangdong Provincial Key Laboratory of Brain Connectome and Behavior, CAS Key Laboratory of Brain Connectome and Manipulation, The Brain Cognition and Brain Disease Institute (BCBDI), Shenzhen Institutes of Advanced Technology, Chinese Academy of Sciences, Shenzhen-Hong Kong Institute of Brain Science-Shenzhen Fundamental Research Institutions, Shenzhen, China

**Keywords:** COVID-19, chest CT, PCR test, pneumonia, diagnosis

## Introduction

The COVID-19 pandemic has become one of the major health crises worldwide (Guan et al., 2020). PCR testing of SARS-CoV-2 is generally used as the gold standard of confirmation for diagnostics of COVID-19. With the growing awareness of the high false-negative rates of PCR testing (Ai et al., 2020; Yang et al., 2020b), whether and which subgroup of patients with initial negative results should be subjected to repeated testing is a matter that requires more clinical data.

Under this circumstance, the value of chest CT as a screening tool in COVID-19 has been vigorously discussed (Ai et al., 2020; Bao et al., 2020; ; Merkus and Klein, 2020). A recent report with 1,014 cases (Ai et al., 2020; Bao et al., 2020) proposed that chest CT has a high sensitivity for the diagnosis of COVID-19 and may be considered as a primary tool for COVID-19 screening. Others have called for caution regarding the usage of CT due to its ionizing radiation, and some have specified its unsuitability as a screening tool for pediatric patients (Merkus and Klein, 2020). After evaluation, our triaging procedure and diagnostic practice for a total of 358 outpatients (149 COVID-19–suspected cases including 13 confirmed) at Shenzhen People’s Hospital, Shenzhen, China, we found that chest CT can distinguish COVID-19 patients from suspected cases (AUC = 0.85) much better than other indicators (AUCs < 0.65) without PCR testing, including epidemiological history, symptoms, white blood cell (WBC) count, and lymphocyte count. Therefore, we propose chest CT as a main tool in determining which subgroup of suspected patients should be subjected to repeated tests after their initial negative PCR testing for SARS-CoV-2.

The first COVID-19 patient was admitted on January 9, 2020 in Shenzhen, an international city with a population of 13.43 million. Different levels of responsive strategies were quickly developed under the pressure of the highly contagious pandemic (Yang et al., 2020a). We received 358 patients at the emergency department and fever clinic with fever or upper respiratory syndromes from January 2020 to March 2020 in our hospital. Among them, 149 were classified as COVID-19–suspected cases according to the criteria complying with the guidelines for the diagnosis and treatment of novel coronavirus pneumonia issued by the National Health Commission of China (NHCC) (National Health Commission and State Administration of Traditional Chinese Medicine, 2020) and required by at least two COVID-19 specialists, and they were subjected to our triaging procedure for suspected cases. Among all 149 patients, after tests and evaluation, 22 patients who were confirmed or highly suspected with COVID-19 were transferred to a special neighboring hospital in Shenzhen that has a special ward for the COVID-19 outbreak.

**FIGURE 1:**
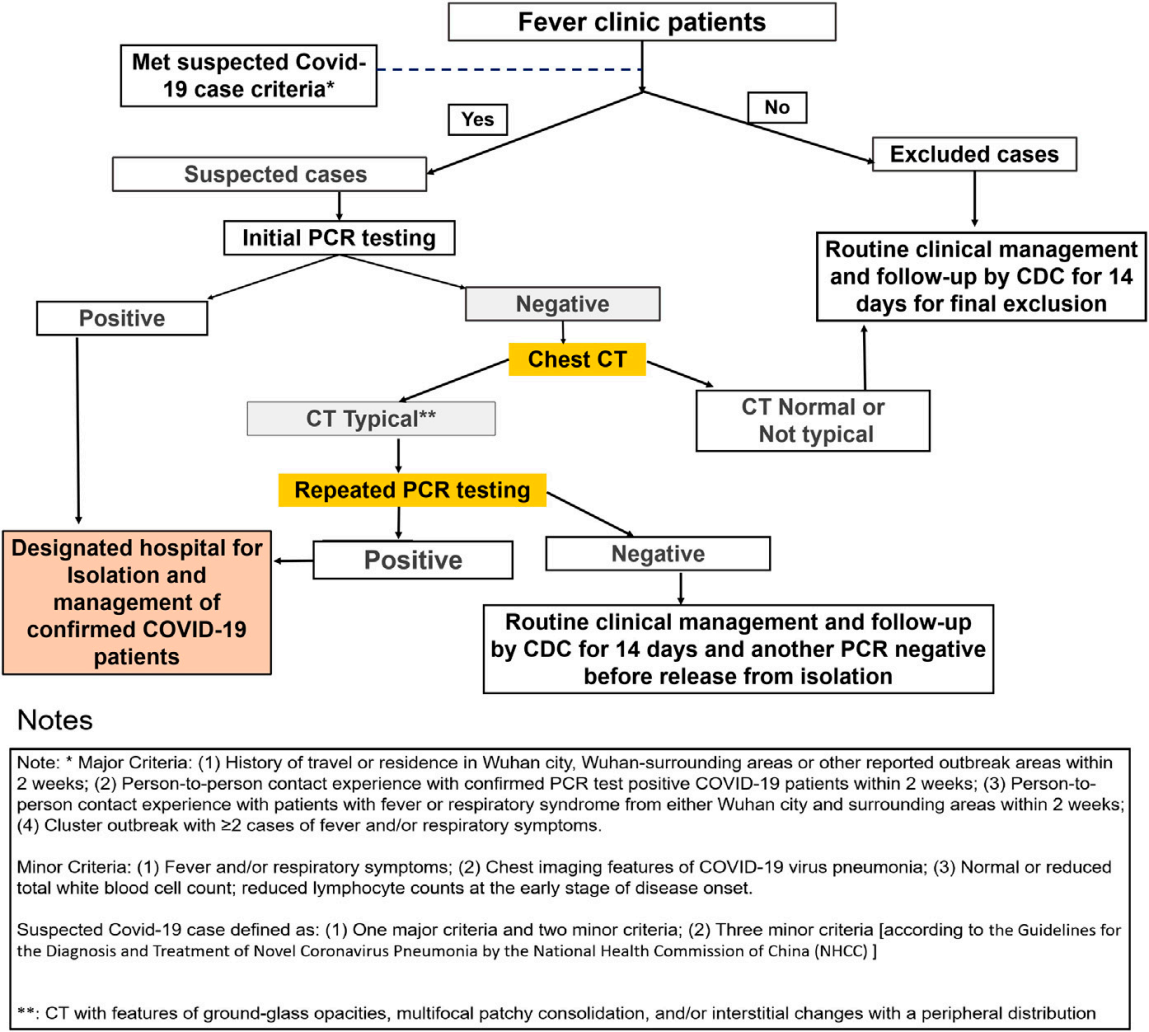
Fever clinic patients were efficiently triaged and diagnosed for COVID-19. The false-negative rate for initial PCR testing is 30.8% (4 out of 13). A high percentage (8.9%) of the initial PCR testing–negative patients with typical CT signature were confirmed to be COVID-19 positive after repeated testing. Initial PCR testing–negative results but chest CT–positive results for viral pneumonia urges for repeated testing for COVID-19 diagnosis.

Our initial oropharyngeal swab PCR testing confirmed 9 SARS-CoV-2-positive patients and 140 negative patients. Expert consultation, chest CT scan, blood WBC counting, epidemiology investigation, and further repeated PCR tests revealed that four patients of the 140 with initial negative PCR tests were false-negative, making the final COVID-19 patient number up to 13. The false-negative initial PCR testing accounts for 30.8% (4 out of 13) of the total confirmed cases (Figure 1), with a true-positive rate of initial PCR test of 69.2% (9 out of 13). This is in line with the reported positive rate from 30 to 60% (Ai et al., 2020) and suggests that additional diagnostic procedures should be carried out after initial negative PCR testing before patients are being subjected to routine clinical follow-up.

## Subsections Relevant for the Subject

### The Description of Four Patients With Positive Computed Tomography and Initial Negative Reverse-Transcription Polymerase Chain Reaction Test

Patient 1: A 35-year-old male with fever for 6 days, with a recent travel history to Wuhan, China, where the pandemic was going on at that time, visited the hospital. Although the initial PCR test for SARS-CoV-2 was negative, the chest CT (January 26, 2020) showed multifocal ground-glass opacity (GGO) and parenchyma consolidation, predominantly involving subpleural regions of both the lungs. A routine blood test showed that the WBC count was 5.24 × 10^9^/L, and the absolute value of the lymphocyte count was 0.87 × 10^9^/L. However, the patient met the diagnostic criteria for a suspected COVID-19 patient and was quarantined at the hospital. The PCR test was repeated with bronchoalveolar lavage fluid (BALF), and the result was positive. Patient 2: A 76-year-old female with fatigue and drowsiness, and with no fever visited the hospital. She had a travel history to Wuhan during the COVID-19 outbreak period. Although the initial PCR test for SARS-CoV-2 was negative, the chest CT (January 23, 2020) showed multifocal parenchyma. A routine blood test showed that the WBC count was 6.96 × 10^9^/L, while the absolute value of the lymphocyte count was 1.09 × 10^9^/L. The second throat swab test was positive. Patient 3: A 66-year-old female appeared with cough and sputum but had no fever. She had a travel history from Wuhan in January 2020. The first swab test was negative. The chest CT (January 25, 2020) showed a bit of GGO. A routine blood test showed that the WBC count was 4.9 × 10^9^/L, and the absolute value of the lymphocyte count was 1.27 × 10^9^/L. A repeated nasopharyngeal swab for the PCR test was positive, and follow-up chest CT showed typical GGO consistent with COVID-19. Patient 4: A 78-year-old male with fever, cough, and sputum, and a recent travel history to Wuhan, China, visited the hospital in January 2020. The first throat swab test was negative. The chest CT (January 25, 2020) showed multifocal GGO. A routine blood test showed that the WBC count was 4.53 × 10^9^/L, and the absolute value of the lymphocyte count was 0.96 × 10^9^/L. However, the patient met the diagnostic criteria for suspected COVID-19 and was quarantined in the hospital. A repeated nasopharyngeal swab for the PCR test was positive.

### Influence of Different Indexes on the Diagnosis of COVID-19

Variable positive rates of the PCR test may be associated with sample collection, PCR methodology, and developing stages of COVID-19 in patients (Ai et al., 2020). Two articles reported using specimens from nasopharyngeal swabs showed a higher positive PCR test rate than oropharyngeal swabs (Tan et al., 2020; Xiao et al., 2020). For the high false-negative rate of the initial PCR test (30.8%), it is necessary to do repeated tests. To avoid occupation of limited hospital resources and speed up the screening procedure, it is not practical to test all the initial PCR-negative patients repeatedly. One parameter or a cluster of test parameters would help in selecting the most possible COVID-19 patients among all the initial PCR test–negative cases.

Typical chest CT refers to ground-glass opacities, multifocal patchy consolidation, and/or interstitial changes with a peripheral distribution (National Health Commission and State Administration of Traditional Chinese Medicine, 2020). The sensitivity and the specificity of typical chest CT in suggesting COVID-19 among all 149 suspected cases are 100% (13/13) and 69.85% (95/136), respectively. A positive WBC parameter means a normal or decreased WBC count (National Health Commission and State Administration of Traditional Chinese Medicine, 2020; National Institutes of Health, 2020). The sensitivity and the specificity of WBC counting in suggesting COVID-19 among all 149 suspected cases are 100% (13/13) and 0.74% (1/136), respectively. The specificity of WBC counting is much lower than that of chest CT. The sensitivity and the specificity of the combination of chest CT positive and WBC counting are 100% (13/13) and 70.59% (96/136), respectively, with not much improvement compared with using chest CT alone as a parameter. When we used the strict parameter combinations in our study according to the guideline of the NHCC, which includes fever/cough, positive result of chest CT manifestations, and WBC counting, along with epidemiology characteristic (history of traveling to Wuhan or being in close contact with COVID-19 patients), the sensitivity and the specificity in suggesting COVID-19 among all suspected cases are 84.62% (11/13) and 88.24% (120/136), respectively. Although the specificity increases from 69.85 to 88.24%, the sensitivity decreases from 100 to 84.62% when compared with using chest CT alone as a parameter.

## Discussion

Among the 140 initial PCR test–negative patients, 95 showed normal chest CT, and they were excluded from the diagnosis of COVID-19 and released after another PCR test confirmation complying with the NHCC guideline. For the 45 cases with pneumonia CT features, 40 were classified as not typical COVID-19 CT presentation, and four were classified as typical COVID-19 CT presentation. In these four cases, repeated PCR tests revealed all positive results (Figure 1), which account for 8.9% (4 out of 45) of the “initial PCR testing negative but chest CT positive for viral pneumonia” group. This is quite a high percentage considering the high contagious nature of SARS-CoV-2. We strongly suggest that for patients with initial PCR testing–negative results but chest CT positive, repeated PCR tests should be carried out. Here we are not considering chest CT as an alternative screening tool to replace the PCR test for COVID-19 but rather proposing that chest CT serves well as a tool in determining which subgroup of suspected patients should be subjected to repeated tests after their negative initial PCR testing for SARS-CoV-2.

Among our 140 initial PCR-negative cases, the sensitivity and the specificity of chest CT in suggesting COVID-19 are 100% (4/4) and 69.85% (95/136), respectively; the sensitivity and the specificity of WBC counting are 100% (4/4) and 0.74% (1/136), respectively; and the sensitivity and the specificity of combined parameters (fever/cough, chest CT, WBC counting, and epidemiology characteristic) are 50.0% (2/4) and 88.24% (120/136), respectively. Considering both the sensitivity and the specificity, chest CT proves to be the most powerful parameter in suggesting COVID-19 among initial PCR-negative cases. In our study, upon using chest CT as a parameter, the repeated PCR testing pressure was reduced by 67.9% (95 out of 145) (Figure 1). All of the CT nontypical cases among the initial negative testing cases were excluded from COVID-19 diagnosis (confirmed by repeat PCR before releasing and then followed up). Altogether, in our study, chest CT serves well as a tool in determining what kind of suspected patients should be subjected to repeated tests after their negative initial PCR testing for SARS-CoV-2.

As demonstrated in Figure 1, our triage operation process showed a high performance, with a screening rate of 14.77% (22/149) in COVID-19–suspected patients to be sent to the special ward, while the screening rate was 0% (0/209) for non-suspected outpatients. For all 149 suspected cases, the top four diagnoses were upper respiratory tract infection (54/149), community-acquired pneumonia (53/149), COVID-19 (13/149), and gastroenteritis (5/149). This suggests that upper respiratory tract infection and community-acquired pneumonia should be emphasized and excluded from the COVID-19 diagnostic process to avoid the occupation of hospital resources. Till date, all 13 confirmed patients have recovered and were discharged.

Our clinical practice showed that with an initial PCR positive rate of 69.2% in those finally confirmed COVID-19 cases, further testing and repeated PCR testing for subgroups of the initial PCR test–negative patients should be emphasized. In a recently published article, it was stated that to compensate for the potential risk of false-negative PCR, chest CT should be applied for clinically suspected patients with negative initial RT-PCR (He et al., 2020), and repeated swab tests should be carried out (Xie et al., 2020). In terms of chest imaging findings, the less pulmonary consolidation found *via* CT scan, the greater the possibility of negative initial RT-PCR results (Chen et al., 2021).

Our study had limitations. First, the limitation of the hospital and the confirmed patient resources is an unavoidable problem for this study. However, from another perspective, as the vaccines are developed and applied, suspected or confirmed patients of COVID-19 infection will be reduced in number. The idea in this opinion article is to somehow provide clinical instruction. Second, the PCR result is affected by many factors, such as laboratory reagents, test methods, and subjective performance. However, at this time, the nucleic acid detection method is not mature yet, and our research results are more important. We try to provide evidence to support the importance of chest CT scan in “work protocol for fever clinic” in detection of COVID-19 patients. In our protocol, chest CT does not serve as a screening tool but as an important indicator for follow-up and repeated PCR testing after initial PCR-negative results. We provide more evidence to show that this work protocol could provide high sensitivity and acceptable specificity, which are better than other criteria and able to reduce the missed diagnosis of false-negative nucleic acid patients.
